# Prädiktive molekulare Diagnostik beim Mammakarzinom: Welche Anforderungen stellen sich heute und in Zukunft für die Pathologie?

**DOI:** 10.1007/s00292-022-01096-y

**Published:** 2022-08-15

**Authors:** Peter J. Wild, Carsten Denkert, C. Jackisch

**Affiliations:** 1 Dr. Senckenbergisches Institut für Pathologie, Universitätsklinikum Frankfurt, Theodor-Stern-Kai 7, 60590 Frankfurt am Main, Deutschland; 2 Institut für Pathologie, Universitätsklinikum Gießen-Marburg, Standort Marburg, Baldingerstraße, 35033 Marburg, Deutschland; 3 Klinik für Gynäkologie und Geburtshilfe, Sana Klinikum Offenbach, Starkenburgring 66, 63069 Offenbach, Deutschland

**Keywords:** Molekularpathologe, Prädiktive Biomarker, Next Generation Sequencing, „Liquid biopsy“, Varianteninterpretation, Pathology, molecular, Predictive biomarkers, Next generation sequencing, Liquid biopsy, Variant interpretation

## Abstract

Mit zunehmenden zielgerichteten Optionen zur Behandlung solider Tumoren wachsen für die Pathologie die Anforderungen an die prädiktive molekulare Diagnostik. Beim Mammakarzinom war das Erfordernis der Bestimmung genomischer prädiktiver Marker für zielgerichtete Therapien bisher überschaubar (Nachweis einer *PIK3CA*-Mutation beim endokrin vorbehandelten Luminaltumor und nur beim sekretorischen Mammakarzinom angezeigte Suche nach *NTRK*-Fusionen). Spätestens bei Nichtansprechen der Erst- bzw. Zweitlinienstandardtherapien ist eine Next-Generation-Sequencing-Panel-Diagnostik sinnvoll, um Resistenzmechanismen z. B. gegen die endokrine Therapie oder „cyclin-dependent kinase 4/cyclin-dependent kinase 6“ (CDK4/CDK6) abzuklären und Ansatzpunkte für in Entwicklung befindliche Therapien zu identifizieren. Die Interpretation sollte qualitätsgesichert gemäß internationalem Standard erfolgen und der interdisziplinären Tumorkonferenz zeitnah in einem transparenten und standardisierten Report zur Verfügung stehen.

## Abstract (en)

With an increasing number of targeted therapy options for the treatment of solid tumors, the demands on predictive molecular diagnostics for pathology are growing. In breast cancer the need to determine genomic predictive markers for available targeted therapies has so far been manageable (detection of PIK3CA mutations in endocrine pretreated luminal tumors and the search for NTRK fusions indicated only in secretory breast cancer). At the latest in cases of nonresponse to first-line or second-line standard treatment, more comprehensive diagnostics using next generation sequencing (NGS) panel diagnostics makes sense. This should be suitable for clarifying resistance mechanisms, e.g. against endocrine therapy or cyclin-dependent kinase 4/cyclin-dependent kinase 6 (CDK4/6) inhibitors and to identify indications for therapies currently in development. The interpretation should be carried out in a quality assured manner in accordance with international standards and the interdisciplinary tumor board should make a transparent and standardized report available in a timely manner.

## Lernziele

Nach der Lektüre dieses Fortbildungsbeitrags wissen Sie …

- welche molekularen Marker beim Mammakarzinom eine Rolle spielen.
- bei welchen therapeutischen Fragestellungen die Bestimmung welcher Biomarker sinnvoll ist.
- worauf es bei der molekularen Diagnostik des Mammakarzinoms ankommt.
- welche Informationen der Pathologiebefundbericht enthalten sollte.
- wie die Ergebnisse der molekularen Diagnostik für die Diskussion im Tumorboard genutzt werden können.

## Einleitung

Moderne **Sequenziertechnologien**

Sequenziertechnologien

haben in den letzten Jahren die Möglichkeiten der Diagnostik von Krebserkrankungen erweitert und zum tieferen Verständnis der Tumorbiologie beigetragen. Auch beim Mammakarzinom sind inzwischen viele krebsrelevante Genveränderungen bekannt [1]. Mit zunehmender Verfügbarkeit von Therapien, die gegen **molekulare Zielstrukturen**

molekulare Zielstrukturen

gerichtet sind, stellt die Diagnostik für die Therapiestratifizierung auch beim Mammakarzinom wachsende Anforderungen an die Pathologie.

Dieser Beitrag gibt einen Überblick über die heute in der klinischen Routine erforderlichen **molekularpathologischen Analysen**

molekularpathologischen Analysen

für die Planung sowie Durchführung der Therapie und zeigt Zukunftsoptionen und Handlungsfelder auf.

## Molekulare Diagnostik für die klinische Routine

Für eine leitliniengerechte Therapie des Mammakarzinoms werden als Grundlage der informierten Behandlungsplanung zunehmend molekularpathologische Analysen benötigt. Aufgrund der Verfügbarkeit von zielgerichteten Therapien beschränkte sich der Bedarf bisher überwiegend auf das metastasierte Mammakarzinom [2]. In Zukunft wird die Bedeutung der **prädiktiven Molekulardiagnostik**

prädiktiven Molekulardiagnostik

zunehmen und auch in frühen Stadien eine Rolle spielen.

**Figure 1:**
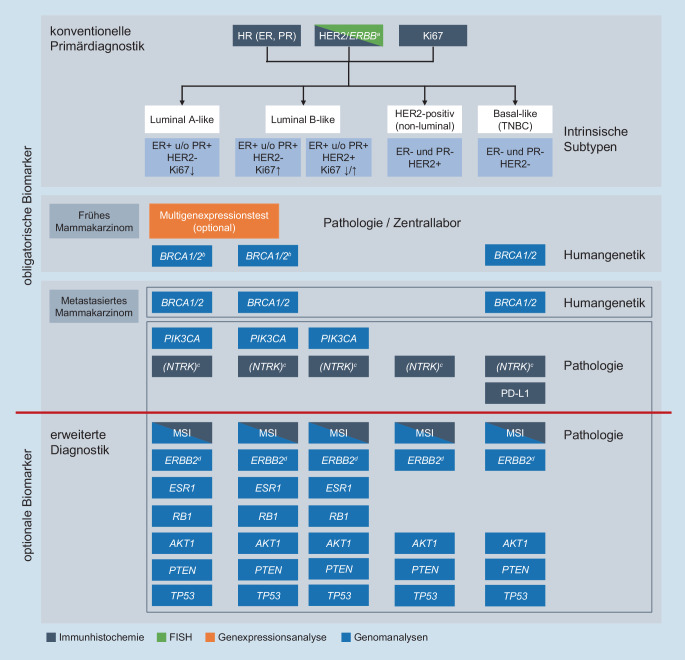

Die **klassische Pathologie**

klassische Pathologie

bildet weiterhin die Grundlage der Diagnostik, sichert die Diagnose und liefert ein deskriptives Bild des Krebsgeschehens. Die Primärdiagnostik und die Einteilung in molekulare Subtypen des Mammakarzinoms erfolgen mithilfe **immunhistochemischer Methoden**

immunhistochemischer Methoden

(Abb. 1). Zielstrukturen für endokrine und etablierte monoklonale Antikörper, die gegen den „human epidermal growth factor receptor 2“ (HER2) gerichtet sind, lassen sich ebenfalls mithilfe konventioneller Methoden nachweisen [3].

Die molekulare Diagnostik ergänzt zunehmend die klassische Pathologie und gibt einen funktionellen Einblick in die Tumorbiologie. So können tumortreibende Mechanismen gezielt identifiziert und ggf. mithilfe zielgerichteter Therapien adressiert werden.

### Prognostische und prädiktive genomische Marker beim frühen Mammakarzinom

Genomanalysen werden bisher beim frühen Mammakarzinom hauptsächlich dann herangezogen, wenn beim hormonrezeptorpositiven HER2-negativen (luminalen) Mammakarzinom der Nutzen einer adjuvanten Chemotherapie anhand klinischer Parameter nicht ausreichend abgeschätzt werden kann (Abb. 1). Für die bessere Beurteilung des individuellen Rückfallrisikos stehen seit einigen Jahren **Multigentests**

Multigentests

zur Verfügung. Diese erfassen die Expression von krebsrelevanten Genen und ermöglichen die quantitative Abschätzung des Rückfallrisikos bzw. den wahrscheinlichen Nutzen einer adjuvanten chemoendokrinen Therapie gegenüber einer alleinigen endokrinen Therapie [3]. Die von der Organkommission Mamma der Arbeitsgemeinschaft für gynäkologische Onkologie (AGO) empfohlenen Tests für pN0‑1-Tumoren sind zu finden unter [2].

Im Rahmen der gesetzlichen Krankenversicherung sind die Multigentests jedoch nur für **nodal-negative Tumoren**

nodal-negative Tumoren

erstattungsfähig. Bei **nodal-positiven N1-Tumoren**

nodal-positiven N1-Tumoren

ist eine Erstattung nur im Rahmen der ambulanten spezialfachärztlichen Versorgung (ASV) oder über Selektivverträge möglich. Ob der Einsatz eines Multigentests für eine Patientin sinnvoll ist, sollte im individuellen Einzelfall nach Abklärung der klinisch-pathologisch relevanten Parameter, die Aufschluss über die Prognose und das individuelle Rezidivrisiko geben können, von der Tumorkonferenz empfohlen werden.

Bisher war die Bestimmung weiterer molekularer Biomarker für die Therapieplanung beim frühen Mammakarzinom nicht gefordert, weil zielgerichtete Therapieoptionen, die diese erfordern würden, für dieses Erkrankungsstadium noch nicht zur Verfügung standen. Eine Ausnahme stellen die Keimbahnanalysen der *BRCA1*‑ und *BRCA2*-Gene (*gBRCA1*/*gBRCA2*) beim **triple-negativen Mammakarzinom**

triple-negativen Mammakarzinom

(TNBC) dar. Die Testung dient zur Abklärung eines möglichen familiären Risikos, war bisher jedoch für die Abschätzung der Wirksamkeit zielgerichteter Therapien nicht relevant (Abb. 1; [3]).

In Zukunft wird der Nachweis einer *gBRCA1-/gBRCA2*-Mutation für die Therapie des HER2-negativen Mammakarzinoms (HR+, HER2 negativ und triple-negativ) therapierelevant werden (Abb. 1). Die Ergebnisse der OlympiA(OLaparib in Adjuvant BRCAm breast cancer)-Studie haben belegt, dass Patientinnen mit frühem HER2-negativem Mammakarzinom und hohem Rezidivrisiko bei Nachweis einer *gBRCA*-Mutation von der adjuvanten Therapie mit dem PARP(Poly ADP-Ribose Polymerase)-Inhibitor **Olaparib**

Olaparib,

der bereits in der metastasierten Situation zugelassen ist, in Form eines verbesserten krankheitsfreien und fernmetastasenfreien Überleben profitieren [8]. Wegen der großen Zahl der infrage kommenden Patientinnen stellen sich in Zukunft Herausforderungen bezüglich der Sicherstellung von ausreichenden Testmöglichkeiten und der zeitnahen Verfügbarkeit der Ergebnisse für die Therapieplanung.

#### Merke

- Multigentests ermöglichen bei unklarem Nutzen einer chemoendokrinen Therapie von Luminaltumoren im Stadium pN0‑1 eine Abschätzung des Rezidivrisikos.
- Bei TNBC wird in frühen Stadien der *gBRCA*-Status bestimmt, um das Vorliegen einer hereditären Erkrankung abzuklären.
- Der Nachweis einer *BRCA1-/BRCA2*-Keimbahn-Mutation wird in Zukunft bei HER2-negativen frühen Karzinomen mit hohem Rezidivrisiko im Zusammenhang mit dem Einsatz eines PARP-Inhibitors, der bei vorliegender Zulassung möglich wird, therapierelevant.

### Molekulare Diagnostik des metastasierten Mammakarzinoms für die klinische Routine

Anders als beim frühen Mammakarzinom, bei dem die prädiktive molekulare Diagnostik, wie oben erwähnt, noch am Anfang steht, zeichnet sich beim metastasierten Mammakarzinom ein Paradigmenwechsel ab. Alterationen in den Genen *BRCA1* und *BRCA2, PIK3CA* und optional *NTRK 1 bis NTRK 3 *haben bereits therapeutische Implikationen (Abb. 1).

#### *BRCA*-Diagnostik

Patientinnen mit HER2-negativem lokal fortgeschrittenem oder metastasiertem Brustkrebs können mit **PARP-Inhibitoren**

PARP-Inhibitoren

behandelt werden, wenn pathogene Mutationen in den Tumorsuppressorgenen *BRCA1* bzw. *BRCA2* nachweisbar sind [9, 10]. In die entsprechenden Phase-III-Studien waren Patientinnen mit BRCA-Keimbahn-Mutationen eingeschlossen. Der Nachweis wird in der Versorgungsrealität auch an Tumorgewebe geführt, da dieses der Pathologie zur Verfügung steht. Ein Nachweis von **Keimbahnmutationen**

Keimbahnmutationen

an Tumorgewebe ist mit einer Konkordanz von 99 % im Vergleich zum Nachweis an Blut möglich [11]. Die Organkommission Mamma der AGO hat die *BRCA*-Analytik im Tumorgewebe als eine „Kann“ (+/−)-Möglichkeit, wenn auch nicht als Regel, eingestuft [2]. Die durchführenden Labore sollten erfolgreich die Qualitätssicherungsmaßnahmen zur BRCA-Testung durchgeführt haben. Auch auf eine qualitätsgesicherte Interpretation der Daten sollte geachtet werden.

Wird eine *BRCA*-Mutation durch die Pathologie am Tumorgewebe detektiert, sollte im Pathologiebericht darauf hingewiesen werden, dass am Tumorgewebe Keimbahnnutationen und **somatische Mutationen**

somatische Mutationen

nachgewiesen, diese aber nicht sicher unterschieden werden können. Beim Mammakarzinom sind etwa ein Drittel der *BRCA*-Mutationen somatisch (*sBRCA*) und zwei Drittel Keimbahnmutationen (*gBRCA*) [12]. Studien am Ovarial- und am Prostatakarzinom belegen ein Ansprechen auf die PARP-Inhibitor-Therapie bei *gBRCA-* und *sBRCA*-Mutation [9]. Die behandelnden Kliniker können bei geplanter PARP-Inhibitor-Therapie des Mammakarzinoms eine weitere Abklärung bezüglich des Vorliegens einer erblichen Genveränderung durch die Humangenetik veranlassen.

Die Testung auf *BRCA-Keimbahn-Mutation* ist in Deutschland technisch etabliert, jedoch zeigte eine Analyse, dass bisher nicht alle Patientinnen, die die Zulassungskriterien für die Therapie mit einem PARP-Inhibitor erfüllen, auf *BRCA*-Mutationen getestet werden. Die Entscheidung zur Testung orientierte sich bisher weitgehend an der **Familienanamnese**

Familienanamnese.

**Figure 2:**
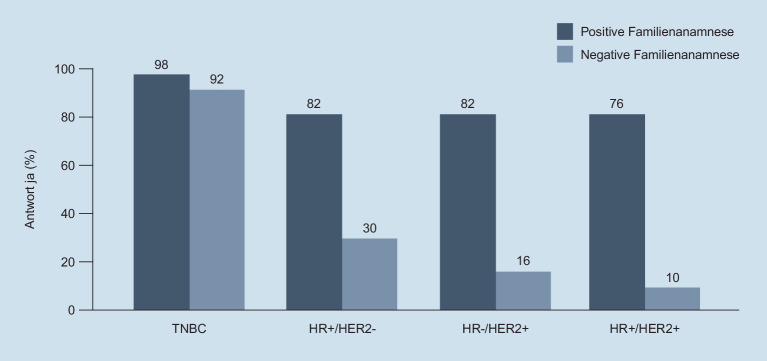

Bei negativer Familienanamnese wurden zwar 92 % der triple-negativen, aber nur 30 % der für die PARP-Inhibitor-Therapie infrage kommenden Patientinnen mit hormonrezeptorpositiven Tumoren getestet (Abb. 2; [13]).

##### Merke

- Beim HER2-negativen metastasierten Mammakarzinom und Nachweis einer *BRCA*-Keimbahn-Mutation (*gBRCA1mt/gBRCA 2mt*) kann eine Therapie mit einem PARP-Inhibitor indiziert sein.
- Wird eine *BRCA*-Mutation durch die Pathologie am Tumorgewebe detektiert, sollte bei geplanter PARP-Inhibitor-Therapie abgeklärt werden, ob eine Keimbahnmutation vorliegt.

#### Nachweis von *PIK3CA*-Mutationen

Beim Mammakarzinom sind Veränderungen im *PIK3CA*-Gen mit etwa 40 % die häufigsten Mutationen [14]. Therapierelevant ist der Nachweis einer *PIK3CA*-Mutation seit Zulassung des PIK3CA-Inhbitors **Alpelisib**

Alpelisib

in Kombination mit dem Östrogenrezeptor(ER)-Antagonisten **Fulvestrant**

Fulvestrant

beim Hormonrezeptor(HR)-positiven endokrin vortherapierten metastasierten Mammakarzinom [15]. Der Nachweis der Wirksamkeit der Kombination wurde im Rahmen der Studie SOLAR‑1 bei Vorliegen von 11 Hotspot-Mutationen in den Exons 7,9 und 11 erbracht [16].

Die Testung erfolgt an Tumorgewebe oder mithilfe des Nachweises an zirkulierender Tumor-DNA (ctDNA) aus einer Plasmaprobe (**„liquid biopsy“**

„liquid biopsy“).

Hierbei ist Folgendes zu beachten:

- Für die Testung an Gewebe sollte möglichst aktuelles Tumorgewebe verwendet werden, falls möglich, ist eine Testung an einer Metastase einer Testung am Primärtumorgewebe vorzuziehen.
- Für die Liquid biopsy müssen Plasmaproben ausreichend freie Tumor-DNA enthalten. Die Pathologie führt dazu nach dem Probeneingang eine Qualitätskontrolle durch. Die Abnahme von Blutproben für die Liquid biopsy muss mithilfe spezieller Röhrchen erfolgen, die die zellfreie DNA stabilisieren. Viele Labore stellen ihren Einsenders diese Abnahmeröhrchen zur Verfügung. Erforderlich sind ein bis 2 Röhrchen à 10 ml Blut. Die Proben sind bei Raumtemperatur bis zu 7 Tage stabil, spätestens dann sollte die Plasmaseparation erfolgen. Wenn mit Ethylendiamintetraessigsäure (EDTA) beschichtete Röhrchen verwendet werden, muss das Plasma sofort separiert werden [17].
- Zur DNA-Extraktion sollten erprobte Standardmethoden angewendet werden. Automatisierte Verfahren reduzieren die Variabilität, können jedoch die DNA-Ausbeute verringern [18].
- Für die Next-Generation-Sequencing(NGS)-basierte Analyse von ctDNA müssen validierte Verfahren mit ausreichender Sensitivität zum Einsatz kommen. Leitlinien für die Validierung blutbasierter Tests wurden publiziert [19].
- Während der Nachweis von *PIK3CA*-Mutationen im Plasma verlässlich ist, sollte ein negatives Testergebnis stets aufgrund der Möglichkeit zu geringer ctDNA-Mengen und niedriger Allelfrequenzen überprüft werden [15]. Der Nachweis von beim Mammakarzinom häufigen (Abb. 2) und in den meisten Tumorpanels abgedeckten *TP53*-Mutationen kann einen Hinweis darauf geben, dass ausreichend ctDNA in der Probe analysiert wurde. Kann der Nachweis nicht geführt werden, sollte zur Verifikation eine Gewebeprobe analysiert werden.

Bei Nachweis einer *PIK3CA*-Mutation in Gewebe oder Plasma muss vom behandelnden Arzt derzeit die Kostenübernahme für Alpelisib beantragt und der Wirkstoff über die internationale Apotheke angefordert werden. Antragsunterlagen sind bei der AGO erhältlich [20]. Patientinnen haben bei Erfüllung der Zulassungskriterien einen Rechtsanspruch auf die Diagnostik und bei Vorliegen einer *PIK3CA*-Mutation auf die entsprechende Therapie. Die Analytik ist für die Pathologie erstattungsfähig.

##### Merke

- Für Patientinnen mit endokrin vorbehandeltem hormonrezeptorpositivem metastasiertem Mammakarzinom kommt bei Nachweis einer *PIK3CA*-Mutation die Therapie mit Alpelisib in Kombination mit Fulvestrant infrage.
- Der Nachweis erfolgt an Gewebe oder an einer Plasmaprobe (Liquid biopsy).
- Kann der Nachweis in der Liquid biopsy nicht geführt werden, sollte eine Gewebeprobe analysiert werden.

#### *NTRK*-Fusionen

Die *NTRK*-Inhibitoren **Larotrectinib**

Larotrectinib

und **Entrectinib**

Entrectinib

sind indikationsübergreifend bei soliden Tumoren mit *NTRK*-Fusionen und lokal fortgeschrittener oder metastasierter Erkrankung zugelassen [21, 22]. Die Genveränderungen sind selten, wird aber eine entsprechende Fusion nachgewiesen, können die Patienten lang anhaltend von der Therapie mit *NTRK*-Inhibitoren profitieren. Die Deutsche Gesellschaft für Hämatologie und Medizinische Onkologie (DGHO) empfiehlt daher in einem Positionspapier die Testung, wenn der Einsatz eines *NTRK*-Inhibitors bei dem jeweiligen Krankheitsbild die beste verfügbare Therapieoption darstellt [23]. Beim Mammakarzinom sind *NTRK*-Fusionen extrem selten und wurden bisher nur beim **sekretorischen Mammakarzinom**

sekretorischen Mammakarzinom

mit relevanter Häufigkeit nachgewiesen. Die Organkommission Mamma der AGO empfiehlt die Diagnostik insbesondere in diesem Tumortyp [2]. Der Nachweis der Fusionen erfolgt meist mithilfe von RNA-basierten Assays oder der **Fluoreszenz-in-situ-Hybridisierung**

Fluoreszenz-in-situ-Hybridisierung

(FISH), ggf. nach immunhistochemischem Vorscreening zum Nachweis der Expression der *NTRK*-Gene 1, 2 oder 3 [24].

##### Merke

- Beim metastasierten sekretorischen Mammakarzinom ist eine Testung auf *NTRK*-Fusionen sinnvoll.
- Der Nachweis sollte möglichst mithilfe geeigneter RNA-basierter NGS-Assays erfolgen.

## Erweiterte molekulare Diagnostik des metastasierten Mammakarzinoms

Beim Mammakarzinom können über die Bestimmung des Status der oben genannten prädiktiven Biomarker hinaus Informationen über mögliche **Resistenzmechanismen**

Resistenzmechanismen

sowie über die potenzielle Wirksamkeit von derzeit in klinischen Studien untersuchten Therapieoptionen gewonnen werden. Die Indikation für eine erweiterte Diagnostik sollte von der Tumorkonferenz gestellt werden, wenn die Therapieplanung umfassende Informationen zur Tumorbiologie erfordert.

### Prädiktive Biomarker für in klinischer Entwicklung befindliche Wirkstoffe

**Figure 3:**
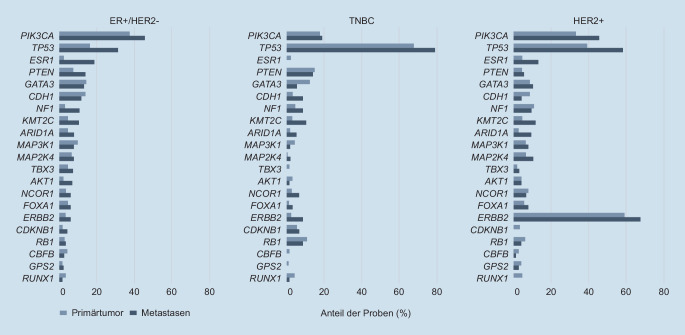

Bei mehr als 40 % der Mammakarzinome konnten in einer Analyse an 625 Tumorproben klinisch relevante genomische Aberrationen nachgewiesen werden [25]. Fehlen Standardtherapieoptionen, können prädiktive Biomarker für potenziell wirksame Therapieansätze und geeignete klinische Studien mithilfe umfassender Panel-Analysen identifiziert werden:

- *ERBB2*-Mutationen, die unabhängig von einer Genamplifikation mit einer Inzidenz bis zu 15 % insbesondere beim invasiven lobulären Mammakarzinom nachgewiesen wurden, können auf eine mögliche Wirksamkeit der Tyrosinkinaseinhibitoren Neratinib bzw. Lapatinib hinweisen [2, 26].
- Bei den in 5,8 % bzw. 12,3 % der Proben detektierten Genveränderungen in *AKT* und *PTEN* können die Proteinkinase-B(AKT)-Inhibitoren Capivasertib oder Ipatasertib den PIK3CA-Signalweg blockieren [27].+
- Der Nachweis einer Mikrosatelliteninstabilität (MSI) bei 1,7 % der Mammakarzinome weist auf die potenzielle Wirksamkeit von Immuncheckpoint-Inhibitoren in dieser kleinen Gruppe hin [2, 28]. Für Pembrolizumab besteht bereits eine Zulassung beim TNBC. Eine Pan-Tumor-Zulassung der U.S. Food and Drug Administration (FDA) als Therapie der letzten Wahl für solide Tumoren mit MSI ermöglicht die Selektion weiterer potenziell profitierender Subtypen [29].
- Inaktivierende *TP53*-Mutationen sind beim metastasierenden Mammakarzinom häufig und können beim luminalen Tumor eine endokrine Resistenz bedingen ([30]; Abb. 3). Derzeit befinden sich verschiedene Substanzen, die die Funktion des Tumorsuppressorgens direkt oder indirekt wiederherstellen, in klinischer Entwicklung [31].

### Hinweise auf primäre oder sekundäre Resistenzen

Whole-Genome-Analysen des primären und des metastasierten Mammakarzinoms zeigen ein unterschiedliches **Mutationsprofil**

Mutationsprofil

(Abb. 3; [25]). Aufgrund des durch die Therapie ausgelösten **Selektionsdrucks**

Selektionsdrucks

finden sich beim endokrin behandelten luminalen Subtyp vermehrt Mutationen in *ESR1*, beim HER-positiven Subtyp insbesondere Mutationen in *ERBB2*.

**Figure 4:**
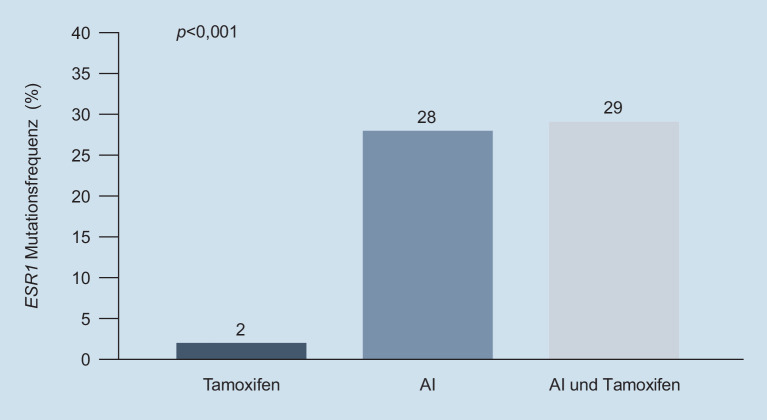

Die *ESR1*-Mutationen, die zu einer ligandenunabhängigen Aktivierung des Östrogenrezeptors führen, sind ein unter Aromatasehemmer im Gegensatz zu Tamoxifen häufig beobachteter Resistenzmechanismus gegen endokrine Therapien (Abb. 4). Als Folgetherapie ist aufgrund des Wirkmechanismus der **selektive Östrogen-Down-Regulator**

selektive Östrogen-Down-Regulator

(SERD) Fulvestrant bei Vorliegen einer *ESR1*-Mutation wirksam. Neue oral verabreichbare SERD befinden sich in klinischer Entwicklung. Neben der *ESR1*-Mutation sind jedoch weitere Mechanismen endokriner Resistenz bekannt, wie beispielweise Mutationen im MAPK-Signalweg [32]. Eine Abklärung durch geeignete Panel-Sequenzierung kann die Wahl der Folgetherapie unterstützen.

Auch eine primäre Resistenz gegen Inhibitoren der „cyclin-dependent kinase 4/cyclin-dependent kinase 6“ (CDK4/CDK6) z. B. aufgrund eines *RB1*-Gen-Verlusts oder des Vorliegens einer *BRCA2-Mutation* könnte so erkannt werden [34, 35]. Spätestens bei Resistenz gegen CDK4-/CDK6-Inhibitoren ist eine umfassende genomische Profilierung sinnvoll, um die Resistenzmechanismen abzuklären, die Therapien entsprechend zu stratifizieren und den Einschluss in klinische Studien zu ermöglichen. Resistenzmechanismen, für die entsprechende internationale Programme zur Verfügung stehen, betreffen den Verlust von *RB1*, aktivierende Mutationen in *AKT1, ERBB2, ESR1, FGFR2, RAS *sowie Amplifikationen von* AURKA und CCNE2 *[36]. Daneben treten unter CDK4-/CDK6-Inhibitor-Therapie gehäuft *PIK3CA-*Mutationen auf, die in der klinischen Routine therapeutisch mit Alpelisib adressiert werden können [34].

Idealerweise sollten Gen-Panel verwendet werden, die die parallele Analyse aller genannten Genveränderungen erlauben. Bei Durchführung solcher umfassenden Analysen wird eine Teilnahme an klinischen Studien oder strukturierten Programmen empfohlen [2].

#### Merke

- In umfassenden Panel-Analysen sollte Tumor-DNA bei entsprechender klinischer Fragestellung auf prädiktive Marker für in Entwicklung befindliche Therapien und Hinweise auf primäre oder sekundäre Resistenzen untersucht werden.
- Der Einschluss der Patienten in klinische Studien oder strukturierte Programme wird empfohlen.

## Methodik für die molekulare Diagnostik des Mammakarzinoms

Die Wahl der verwendeten Technologien für die definitiven molekulargenetischen Analysen richtet sich nach der jeweiligen klinischen Fragestellung unter Berücksichtigung der Kosteneffizienz. Grundsätzlich stehen Einzelgentests und die umfassende Sequenzierung vieler Genbereiche bis hin zum Gesamtgenom mithilfe des NGS zur Verfügung.

Zum Nachweis einer *BRCA*-Mutation sind **Next-Generation-Sequencing-basierte Methoden**

Next-Generation-Sequencing-basierte Methoden

erforderlich, weil Genveränderungen ohne Konzentration auf **Hotspot-Regionen**

Hotspot-Regionen

in allen Exons der sehr großen Gene *BRCA1* und *BRCA2* vorliegen können [37]. Für die Pathologie ist bei klinischer Relevanz einer umfassenden Diagnostik die Verwendung von geeigneten Gen-Panels, die die *BRCA*-Gene einschließen, sinnvoll. Zur alleinigen Indikationsstellung einer PARP-Inhibitor-Therapie ist, wie oben erwähnt, der Nachweis einer Keimbahnmutation durch Labore der Humangenetik oder des Konsortiums für familiären Brust- und Eierstockkrebs erforderlich. Liegt eine positive Familienanamnese für hereditären Brust- und Eierstockkrebs vor, untersucht das Konsortium zusätzlich zur *BRCA*-Analytik weitere Gene, um das Risiko eines Zweitkarzinoms bei der Patientin und das Erkrankungsrisiko für Familienangehörige umfassend abzuklären [38].

Der Nachweis einzelner bekannter somatischer Genveränderungen in Hotspot-Regionen wie z. B. von *PIK3CA*-Mutationen ist mithilfe von Polymerase-Kettenreaktion(PCR)-Einzelgentests, die **mutationsspezifische Sonden**

mutationsspezifische Sonden

verwenden, kostengünstig und mit ausreichend hoher Sensitivität möglich. Es können jedoch nur bekannte Genveränderungen in definierten Hotspot-Regionen von *PIK3CA *nachgewiesen werden; seltene Genveränderungen oder solche in weiteren Genen werden mit dieser Methode übersehen. Darüber hinaus ist eine separate Tumorprobe für jeden Test erforderlich. Die meisten Labore verwenden in der klinischen Routine kommerziell erhältliche oder Custom-NGS-Gen-Panel unterschiedlicher Größe, die die *PIK3CA*-Mutationen einschließen und weitere relevante tumorgenetische Veränderungen größtmöglich abdecken. Die Gen-Panel müssen dazu vom Pathologielabor für die lokale Nutzung validiert werden.

### Merke

- Zur umfassenden Abklärung der Tumorbiologie des Mammakarzinoms sind NGS-basierte Analysen erforderlich.
- Für die lokale Pathologie bietet sich die Nutzung von Assays an, die die Analyse auf alle therapierelevanten tumorgenetische Veränderungen ermöglichen.

### Wachsende Herausforderungen an die Interpretation NGS-basierter Analysen

Die Interpretation komplexer NGS-Analysen stellt eine wachsende Herausforderung für die Molekularpathologie dar. Um die Bedeutung detektierter Genveränderungen einschätzen und diese qualitätsgesichert berichten zu können, sind geeignete Technologien und personelle Expertise erforderlich. Die korrekte Klassifizierung detektierter Varianten entsprechend internationaler Standards ist essenziell und erfordert bereits z. B. bei der alleinigen *BRCA*-Diagnostik die sorgfältige Recherche in verschiedenen öffentlich zugänglichen **Datenbanken**

Datenbanken

[37, 39]. Werden zunehmend größere Gen-Panel analysiert, kann die Bedeutung neuer Genvarianten erst nach umfassender Recherche oder auch gar nicht zugeordnet werden. Zur Beurteilung der klinischen Relevanz detektierter Varianten sollten transparente **Algorithmen**

Algorithmen

wie beispielweise der AMP(Association for Molecular Pathology)/CAP(College of American Pathologists) oder ESCAT (European Society for Medical Oncology Scale for Clinical Actionability of molecular Targets) Score angewendet werden; diese ermöglichen eine abgestufte Zuordnung der klinischen Evidenz für infrage kommende Therapieoptionen [40, 41].

Zunehmend kommen **intelligente Software-Lösungen**

intelligente Software-Lösungen

zum Einsatz. Diese führen die zeitraubenden Recherchen in Datenbanken automatisiert durch, unterstützen die klinische Interpretation und stellen die Aktualität der dem Bericht zugrunde liegenden Informationen sicher [42, 43, 44]. Relevant sind die Integrierbarkeit in lokale Laborprozesse, eine regelmäßige und verlässliche qualitätsgesicherte Aktualisierung der verwendeten Datenbanken und eine nachvollziehbare Auswertung zur klinischen Relevanz der Ergebnisse.

Für die befunderstellende Pathologie müssen bei Verwendung kommerzieller Software die Schritte von der Variantenannotation bis zur klinischen Interpretation transparent und nachvollziehbar sein. Wenn verfügbar, sollten CE-gekennzeichnete Produkte genutzt werden. Solche Softwarelösungen können über die Unterstützung der Tumorboards bei der Therapieselektion hinaus auch zur zentralisierten Datensammlung beitragen.

#### Merke

- Die korrekte Klassifizierung detektierter Varianten entsprechend internationaler Standards ist essenziell.
- Zur Beurteilung der klinischen Relevanz detektierter Varianten sollten transparente Algorithmen wie beispielweise der AMP/CAP- oder ESCAT-Score angewendet werden.
- Innovative Software-Lösungen können die Variantenannotation bis zur klinischen Interpretation automatisiert durchführen. Wenn verfügbar, sollten CE-gekennzeichnete Produkte genutzt werden.

### Anforderungen an den molekularpathologischen Befund

Der Bericht zum molekularpathologischen Befund muss umfassend über die verwendete Methode, ihre Sensitivität, die Interpretationsgrundlagen sowie die Ergebnisse und deren Interpretation Aufschluss geben. Im Einzelnen sind folgende Angaben erforderlich:

- Patientendaten, einschließlich Diagnose,
- Tumormaterial und Tumorzellgehalt,
- angewendete Sequenziermethode, einschließlich Sensitivität,
- analysierte Zielsequenzen, verwendete Referenzsequenz,
- Variantenallelfrequenz und Sequenziertiefe,
- Angabe der Genveränderungen nach internationalen Vorgaben,
- klinische Relevanz der detektierten Varianten mit funktionellen Konsequenzen und Hinweisen auf potenziell wirksame Substanzklassen mit zugelassenen zielgerichteten Therapien sowie Evidenz für Off-label-Therapien und geeignete idealerweise lokal rekrutierende klinische Studien.

Eine Standardisierung der Befunde durch interne oder kommerzielle in die interne Infrastruktur integrierbare Software-Lösungen kann die folgende interdisziplinäre Zusammenarbeit erleichtern.

### Therapieentscheidung auf der Grundlage molekularer Analysen

Damit Therapieentscheidungen auf der Grundlage des molekularpathologischen Befunds zeitnah getroffen werden können, benötigt die Tumorkonferenz rechtzeitig vollständige Ergebnisse und Informationen zu ihrer klinischen Relevanz. Ergebnisse mit fraglicher klinischer Relevanz sollten im molekularen Tumorboard diskutiert werden. Folgende Faktoren sind relevant, damit Ergebnisse von NGS-Analysen optimal für die Therapieplanung genutzt werden können:

1. Eine Sequenzierung in der lokalen Pathologie ist bei Verfügbarkeit und Abrechenbarkeit der entsprechenden Methodik der Versendung an Zentrallabore vorzuziehen.
2. Qualitätssicherungsmaßnahmen für die angewendeten Technologien zur Sequenzierung und zur Dateninterpretation müssen implementiert sein.
3. Die Analysedaten und die sich daraus ableitenden Ergebnisse müssen den Teilnehmern des Tumorboards zugänglich sein, und die Evidenzgrundlage für die klinische Interpretation muss aufzeigt werden.
4. Der Befund muss über potenziell wirksame Therapieoptionen, einschließlich des Zulassungsstatus und der zugrunde liegenden Evidenz, sowie potenziell geeignete lokal rekrutierende klinische Studien informieren.

Die anschließende Therapieempfehlung auf Grundlage des molekularpathologischen Befunds sollten nachvollziehbaren und von der Tumorkonferenz definierten Algorithmen folgen.

## Fazit für die Praxis

- Für eine leitliniengerechte Therapie des Mammakarzinoms werden zunehmend molekularpathologische Analysen benötigt.
- Von der Pathologie wird mindestens der Nachweis von Alterationen in den Genen *PIK3CA* und ggf. *NTRK* für zugelassene zielgerichtete Therapie abgefordert.
- Durch eine erweiterte molekulare Diagnostik können darüber hinaus mögliche Resistenzmechanismen, prädiktive Marker für in Entwicklung befindliche Therapien sowie potenziell geeignete klinische Studie identifiziert werden.
- Intelligente Softwarelösungen können die Interpretation molekular-pathologischer Analysen unterstützen und die interdisziplinäre Zusammenarbeit erleichtern.
- Im Befundbericht muss umfassend über die verwendete Methode, ihre Sensitivität, die Interpretationsgrundlagen sowie die Ergebnisse und deren Interpretation Aufschluss gegeben werden.
- Molekulare Tumorboards ermöglichen die interdisziplinäre Diskussion von Fällen mit fraglicher klinischer Konsequenz der Analyseergebnisse.

## CME-Fragebogen

Bei welcher Fragestellung kommen bei diagnostiziertem frühem Mammakarzinom Multigenexpressionstests infrage?

Entscheidung für oder gegen endokrine Therapie

Abschätzung des Rezidivrisikos bei unklarem Nutzen einer chemoendokrinen Therapie bei Luminaltumoren im Stadium pN0‑1

Wahl der adjuvanten Chemotherapie, abhängig vom Rezidivrisiko

Genomische Profilierung zur Einbindung in klinische Studien für das metastasierte Mammakarzinom

Bestimmung von Resistenzmarkern gegen Inhibitoren der „cyclin-dependent kinase 4/cyclin-dependent kinase 6“ (CDK4-/CDK6)

Der Status welcher genomischen Marker für zugelassenen Therapien ist beim Mammakarzinom therapierelevant?

Somatische *BRCA*-Mutation und *PIK3CA*-Mutation

Somatische *BRCA*-Mutation und *NTRK*-Fusion

*PIK3CA*-Überexpression und *NTRK*-Fusion

*BRCA*-Keimbahn-Mutation und somatische *BRCA*-Mutation

*BRCA*-Keimbahn-Mutation und *PIK3CA*-Mutation

Wie ist durch die Tumorkonferenz zu verfahren, wenn bei Paneldiagnostik an Tumorgewebe eine *BRCA*-Mutation detektiert wurde und für die betreffende Patientin eine PARP-Inhibitor-Therapie vorgesehen ist?

Wiederholung der Testung an „zirkulierender Tumor-DNA“ (ctDNA; „liquid biopsy“)

Einleitung einer zusätzlichen endokrinbasierten Therapie

Es sollte durch eine Testung im Blut abgeklärt werden, ob eine Keimbahnmutation vorliegt

Keine weiteren Maßnahmen

Einleitung einer zusätzlichen Therapie mit Inhibitoren der „cyclin-dependent kinase 4/cyclin-dependent kinase 6“ (CDK4-/CDK6)

Wie viele Mammakarzinompatientinnen mit fortgeschrittenem Mammakarzinom haben einen *PIK3CA*-mutierten Tumor?

Etwa 5 %

Etwa 10 %

Etwa 40 %

Etwa 70 %

Etwa 90 %

Wie ist zu verfahren, wenn in der „liquid biopsy“ bei geplanter Therapie mit Alpelisib *keine PIK3CA*-Mutation nachgewiesen wurde?

Es sollte eine Gewebeprobe analysiert werden.

Wiederholung der Liquid biopsy an derselben Plasmaprobe

Analyse einer Blutprobe auf Keimbahnmutation

Durchführung einer Liquid biopsy mit Einsatz der doppelten Probenmenge

Erneute Blutabnahme für eine Liquid biopsy

Bei welchem Typ des Mammakarzinoms kommen *NTRK*-Fusionen mit relevanter Häufigkeit vor?

Inflammatorisches Mammakarzinom

Medulläres Mammakarzinom

„Human-epidermal-growth-factor-receptor-2“(HER2)-positives nonluminales Mammakarzinom

Invasiv-duktales Mammakarzinom

Sekretorisches Mammakarzinom

Bei der NGS-Panel-Analyse eines hormonrezeptorpositiven metastasierten Mammakarzinoms wurde im Gewebe eine *ESR1*-Mutation nachgewiesen. Welche Informationen sollten in der Tumorkonferenz übermittelt werden?

Resistenz durch Mutation des Östrogenrezeptors gegen jede weitere endokrine Therapie

Resistenz gegen Inhibitoren der „cyclin-dependent kinase 4/cyclin-dependent kinase 6“ (CDK4-/CDK6)

Gesteigerte Sensitivität gegenüber Folgetherapie mit einem „selective estrogen receptor degrader“ (SERD) wie Fulvestrant

Gesteigerte Sensitivität gegenüber Aromatasehemmer

Gesteigerte Sensitivität gegenüber Tamoxifen

Welche Bedeutung hat der Nachweis einer aktivierenden *ERBB2*-Mutation beim metastasierten Mammakarzinom?

Potenzielle Wirksamkeit von Tyrosinkinaseinhibitoren

Indikation für Trastuzumab

Potenzielle Wirksamkeit von Pembrolizumab

Potenzielle Wirksamkeit endokriner Therapien

Indikation für „selective estrogen receptor degraders“ (SERD) wie Fulvestrant

Welche Bedeutung kann der Nachweis des *RB1*-Gen-Verlustes beim Mammakarzinom haben?

Gesteigerte Sensitivität gegenüber Aromatasehemmer

Gesteigerte Sensitivität gegenüber Tamoxifen

Resistenz gegen jede weiter endokrine Therapie

Resistenz gegen Therapie mit Inhibitoren der „cyclin-dependent kinase 4/cyclin-dependent kinase 6“ (CDK4-/CDK6)

Potenzielle Wirksamkeit von Inhibitoren der Proteinkinase B (AKT)

Welche Konsequenzen für die Therapieplanung ergeben sich bei Nachweis einer Mikrosatelliteninstabilität beim Mammakarzinom?

Potenzielle Wirksamkeit von Immuncheckpoint-Inhibitoren

Hinweis auf Resistenz gegen Immuncheckpoint-Inhibitoren

Erhöhte Sensitivität gegenüber taxanhaltiger Chemotherapie

Resistenz gegenüber taxanhaltiger Chemotherapie

Resistenz gegen endokrinbasierte Therapie
